# Possible Effects of Changes in Carbonate Concentration and River Flow Rate on Photochemical Reactions in Temperate Aquatic Environments

**DOI:** 10.3390/molecules28207072

**Published:** 2023-10-13

**Authors:** Davide Vione, Federica Saglia, Carola Pelazza

**Affiliations:** Department of Chemistry, University of Torino, Via Pietro Giuria 5, 10125 Torino, Italy; federica.saglia@edu.unito.it (F.S.); carola.pelazza@edu.unito.it (C.P.)

**Keywords:** direct and sensitised photolysis, pollutant fate, contaminants of emerging concern, pharmaceuticals and personal care products, non-steroidal anti-inflammatory drugs

## Abstract

In temperate environments, climate change could affect water pH by inducing enhanced dissolution of CaSO_4_ followed by biological sulphate reduction, with the potential to basify water due to H^+^ consumption. At the same time, increased atmospheric CO_2_ could enhance weathering of carbonate rocks (e.g., dolomite) and increase the total concentration of dissolved carbonate species. Both processes enhance phototransformation by the carbonate radical (CO_3_^•−^), as shown for the non-steroidal anti-inflammatory drug paracetamol, provided that the dissolved organic carbon of water does not undergo important fluctuations. Climate change could also affect hydrology, and prolonged drought periods might considerably decrease flow rates in rivers. This is a substantial problem because wastewater pollutants become less diluted and, as a result, can exert more harmful effects due to increased concentrations. At the same time, in low-flow conditions, water is also shallower and its flow velocity is decreased. Photochemical reactions become faster because shallow water is efficiently illuminated by sunlight, and they also have more time to occur because water takes longer to cover the same river stretch. As a result, photodegradation of contaminants is enhanced, which offsets lower dilution but only at a sufficient distance from the wastewater outlet; this is because photoreactions need time (which translates into space for a flowing river) to attenuate pollution.

## 1. Introduction

The contaminants of emerging concern (CECs) are a significant threat to surface water bodies because of their adverse effects, even at low concentration values, on aquatic life forms and potentially on human health as well, if the water is used for irrigation purposes, recreational activities, or as a source of drinking water [1,2]. CECs are often polar and biorecalcitrant compounds, which makes their attenuation difficult in traditional wastewater treatment plants (WWTPs). The activated sludge step of WWTPs is often ineffective in CEC removal, because biorecalcitrance prevents biodegradation while polarity inhibits partitioning onto the sludge [3,4]. Moreover, CECs can reach surface waters from sources other than WWTPs, such as groundwater, irrigation water, untreated wastewater, and human skin (e.g., solar filters during swimming and sunbathing) [2,3].

An important class of CECs is represented by pharmaceuticals and personal care products, of which non-steroidal anti-inflammatory drugs are among the medicines most used by the general population and are often found in surface waters [3,5]. The biological elimination of biorecalcitrant CECs from surface-water environments is usually as problematic as it is in WWTPs, thus abiotic processes play an important role in the attenuation of these compounds. Abiotic processes include hydrolysis, partitioning to other phases (e.g., sediments or the gas phase) that are possibly followed by transformation [6], and aqueous-phase photodegradation [7].

In particular, photochemical attenuation of CECs in surface waters involves the direct photolysis of sunlight-absorbing compounds (where sunlight absorption by CECs triggers transformation), as well as reaction with photochemically produced reactive intermediates (PPRIs) [8,9]. PPRIs are generated upon absorption of sunlight by naturally occurring photosensitisers (e.g., nitrate, nitrite, and chromophoric dissolved organic matter (CDOM)), and they include the hydroxyl (^•^OH) and carbonate (CO_3_^•−^) radicals and singlet oxygen (^1^O_2_), as well as the excited triplet states of CDOM (^3^CDOM*) [10]. PPRIs are quickly eliminated from surface waters, the main processes being scavenging by DOM (dissolved organic matter, not necessarily chromophoric), bicarbonate, and carbonate (in the case of ^•^OH); scavenging by DOM alone (CO_3_^•−^); quenching by dissolved oxygen (^3^CDOM*); and collision with water (^1^O_2_) [11,12]. Because of the production/elimination budget, PPRIs usually reach low steady-state concentrations in sunlit surface waters (around 10^−16^ M for ^•^OH, 10^−14^ M for CO_3_^•−^, and 10^−15^ M for ^3^CDOM* and ^1^O_2_) [8]. Note that PPRIs can be very important in the removal of CECs, but reaction with CECs is usually a minor scavenging process for PPRIs [11].

The rates of aquatic photochemical reactions mainly depend on sunlight irradiance and spectrum, water depth, and water chemistry. Depth is important because shallow waters are better illuminated by sunlight than deep waters, while water chemistry determines the importance of different photosensitisers and scavengers/quenchers in photoreactions [8]. Furthermore, surface-water photochemistry is also affected by some hydrology-related phenomena such as mixing vs. stratification of lake water, evaporative water concentration, and flow rate in rivers [11]. In the latter case, water scarcity has potential to induce faster photoreactions because it decreases depth, which accelerates photoinduced processes. Water velocity is also decreased and, because water travels more slowly in a given river stretch [13,14], this provides more time for faster photoreactions to occur.

Climate change has strong potential to affect photoreactions by modifying both water chemistry and hydrology [11]. In boreal environments, such as the Scandinavian peninsula, climate change favours the phenomenon of browning (increased coloration of surface waters due to enhanced export of CDOM from the surrounding basin) due to increasing average precipitation and a higher frequency of extreme precipitation events [15,16,17,18]. Browning affects, among others, the features of lake-water stratification during summer and it modifies photoreaction pathways by enhancing processes induced by ^3^CDOM* and ^1^O_2_, as well as by inhibiting reactions involving ^•^OH, CO_3_^•−^, and the direct photolysis [11].

In temperate environments, climate change can induce modifications in pH, alkalinity, and inorganic carbon concentrations in surface waters [11,19], and it affects water flow in rivers through a variable precipitation regime [13,20]. These phenomena have the potential to modify photoreaction pathways and kinetics [11]. The present work aims at investigating the photochemical implications of changes in pH/inorganic carbon, as well as water flow that can be induced by climate change in temperate areas. The effects are addressed by means of a modelling approach that makes use of software (APEX: Aqueous Photochemistry of Environmentally occurring Xenobiotics) to understand the role of environmental changes in the photoinduced attenuation processes of CECs. In particular, APEX computes steady-state concentrations of PPRIs and first-order photodegradation rate constants of contaminants on the basis of the sunlight irradiance/spectrum, water chemistry, and water depth [21]. A first-order kinetic model is used by APEX because PPRIs are in steady-state (second-order reactions can thus be treated with the much simpler pseudo-first-order formalism) and because, in almost all conditions, contaminants are negligible PPRI scavengers (which rules out zero-order kinetics).

**Figure 1:**
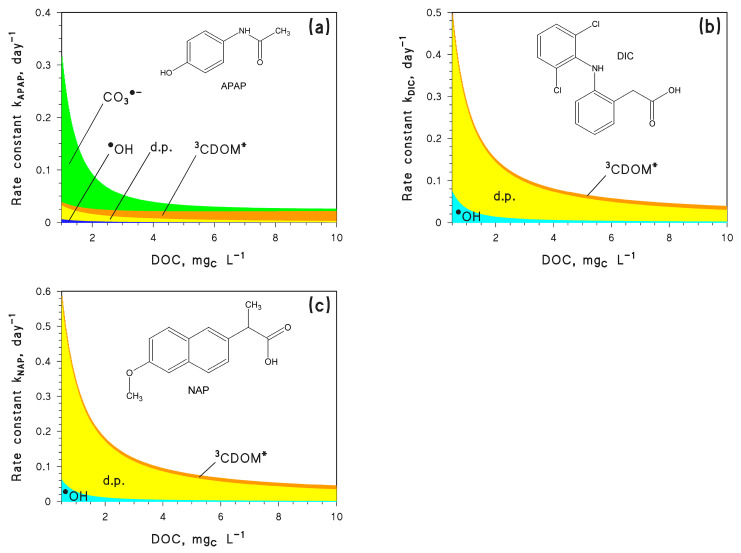
Modelled photoreaction kinetics and pathways for the contaminants under study, as a function of the dissolved organic carbon (DOC): (**a**) Paracetamol (APAP), (**b**) Diclofenac (DIC), and (**c**) Naproxen (NAP). Other water conditions: 3 m depth, 10^−4^ M NO_3_^−^, 10^−6^ M NO_2_^−^, 10^−3^ M HCO_3_^−^, and 10^−5^ M CO_3_^2−^. Sunlight irradiance as per fair-weather spring equinox noon at mid latitude. Note that d.p. = direct photolysis. The different photodegradation pathways (direct photolysis and reaction with ^•^OH, CO_3_^•−^, and ^3^CDOM*) are highlighted with different colours.

As CECs, we here considered three different non-steroidal anti-inflammatory drugs: paracetamol (N-(4-hydroxyphenyl)acetamide, also known as acetaminophen, hereinafter APAP), diclofenac (2-[2-(2,6-dichloroanilino)phenyl]acetic acid, DIC), and naproxen ((2S)-2-(6-Methoxy-2-naphthyl)propanoic acid, NAP). These compounds were chosen because they all undergo fast photodegradation. Furthermore, they follow different photoreaction pathways because DIC and NAP are mainly degraded by direct photolysis [21], while APAP mainly undergoes indirect photochemistry especially by CO_3_^•−^ reactions (Figure 1) [21]. Furthermore, APEX was able to predict the photoreaction kinetics of both DIC and NAP in the epilimnion of Lake Greinfensee (Switzerland), as reported in a field study (lifetimes of 8 and 14 days, respectively) [22], which suggests that model predictions of photodegradation of these compounds would be reasonably accurate. The photodegradation of two compounds undergoing acid⇆base equilibria (sunlight filter benzophenone-4, pKa~7.5 and antibacterial triclosan, pKa~8 [21]) was also considered, in order to obtain insight into the additional effects of pH variations.

The main novelty of this work is the consideration of the photochemical impact of changes that will increasingly affect surface-water environments in the near future.

## 2. Results and Discussion 

### 2.1. Changes in pH and Total Carbonates

Modifications of pH values, as well as *C*_tot_ = [H_2_CO_3_] + [HCO_3_^−^] + [CO_3_^2−^], have the potential to primarily affect processes induced by CO_3_^•−^, which is produced by oxidation of inorganic carbon species [23,24]. In particular, oxidation of CO_3_^2−^ is faster than that of HCO_3_^−^, while H_2_CO_3_ is practically unreactive. In addition to CO_3_^•−^ formation, variations in pH and *C*_tot_ would also affect the scavenging of ^•^OH [25]:

HCO_3_^−^ + ^•^OH → CO_3_^•−^ + H_2_O [*k*_1_ = 8.5 × 10^6^ M^−1^ s^−1^] (1)

CO_3_^2−^ + ^•^OH → CO_3_^•−^ + OH^−^ [*k*_2_ = 3.9 × 10^8^ M^−1^ s^−1^] (2)

The pH value of surface-water bodies can be affected by enhanced dissolution of CaSO_4_ in warmer water, followed by biological reduction of SO_4_^2−^ into organic sulphur [11,19]:

CaSO_4 (s)_ ⇆ Ca^2+^ + SO_4_^2−^ (3)

SO_4_^2−^ + R-H + 8 H^+^ + 6 e^−^ → R-SH + 4 H_2_O (4)

Semireaction 4 consumes H^+^ and, because most electron donors exchange H^+^ and e^−^ in 1:1 ratio, the reduction process of SO_4_^2−^ to R-SH has the potential to consume 2 H^+^ for every processed SO_4_^2−^ ion, thereby inducing a pH increase. This is one of the possible reasons for the observed long-term increase in alkalinity and pH in lake water; the other reason is recovery from acidification, due to lesser atmospheric pollution in regions where the acidity of rainwater is progressively decreasing [26].

The APEX software was here used, first to assess the steady-state [^•^OH] and [CO_3_^•−^] as a function of pH, and then to predict the photodegradation kinetics of APAP. This compound was chosen because reaction with CO_3_^•−^ accounts for over 50% of APAP photodegradation, at least for DOC < 4 mg_C_ L^−1^ (Figure 1a). Photochemical modelling was carried out by assuming water depth *d* = 3 m, DOC = 1 mg_C_ L^−1^, 10^−4^ M NO_3_^−^, 10^−6^ M NO_2_^−^, and 10^−3^ M total inorganic carbon (*C*_tot_), which are reasonable values for surface freshwaters [11]. The irradiance of sunlight (22 W m^−2^ at 290–400 nm), here and in all following calculations, corresponds to fair-weather midday on the spring equinox at mid latitude.

The concentration values of the three carbonate species as a function of pH and *C*_tot_ are expressed as follows [27]:

(5) $${[H}_{2}{\mathrm{CO}}_{3}]=\frac{10^{-2\;\mathrm{pH}}}{10^{-2\;\mathrm{pH}}+K_{a1}\;10^{-\;\mathrm{pH}}+K_{a1}\;K_{a2}}C_{\mathrm{tot}}$$

(6) $${[\mathrm{HCO}}_{3}^{-\;}]=\frac{K_{a1}\;10^{-\;\mathrm{pH}}}{10^{-2\;\mathrm{pH}}+K_{a1}\;10^{-\;\mathrm{pH}}+K_{a1}\;K_{a2}}C_{\mathrm{tot}}$$

(7) $${[\mathrm{CO}}_{3}^{2-\;}]=\frac{K_{a1}\;K_{a2}}{10^{-2\;\mathrm{pH}}+K_{a1}\;10^{-\;\mathrm{pH}}+K_{a1}\;K_{a2}}C_{\mathrm{tot}}$$

where [H^+^] = 10^−pH^, *K*_a1_ = 5 × 10^−7^, and *K*_a2_ = 5 × 10^−11^.

**Figure 2:**
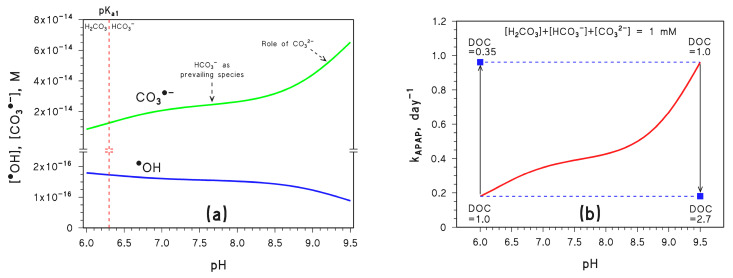
(**a**) Trend with pH of the steady-state [CO_3_^•−^] and [^•^OH]. Note the break in the Y-axis; the vertical dashed line highlights the pK_a_ value of H_2_CO_3_. (**b**) Trend with pH of the first-order photodegradation rate constant of APAP (*k*_APAP_); the DOC units are [mg_C_ L^−1^]. Conditions in both cases: 3 m depth, 10^−4^ M NO_3_^−^, 10^−6^ M NO_2_^−^, *C*_tot_ = 10^−3^ M, DOC = 1 mg_C_ L^−1^ (unless otherwise stated), sunlight irradiance as per fair-weather spring equinox noon at mid latitude. Different curve colours are just for readability. The blue squares represent the impact of DOC variations on *k*_APAP_, the vertical arrows highlight the DOC changes, and the horizontal dashed lines are guides for the eye.

Figure 2a shows the steady-state [^•^OH] and [CO_3_^•−^] calculated by APEX, as a function of pH in the above conditions. Figure 2b shows the corresponding value of the pseudo-first-order photodegradation rate constant *k*_APAP_.

As can be seen in Figure 2a, the steady-state [CO_3_^•−^] increases with increasing pH. Below pH 7, the reason is the lack of significant reactivity between H_2_CO_3_ and ^•^OH, combined with the ability of ^•^OH to oxidise HCO_3_^−^ to CO_3_^•−^ (reaction 1) [25]. As a consequence, the increasing fraction of bicarbonate that occurs as pH increases enhances the formation rate of CO_3_^•−^; at the same time, higher bicarbonate accelerates ^•^OH scavenging and accounts for decreasing [^•^OH]. The almost constant [CO_3_^•−^] at pH 7–8 is accounted for by the fact that bicarbonate is the main inorganic carbon species reacting with ^•^OH in such pH interval to produce CO_3_^•−^, while increasing [CO_3_^•−^] above pH 8 is due to the increasing role of carbonate in CO_3_^•−^ production.

In the latter case, the reason is a combination of the higher reactivity with ^•^OH of carbonate compared to bicarbonate (reactions 1,2) [25] and of oxidation by ^3^CDOM* that is possible with carbonate only (reaction 8) [23,24]. Scavenging of ^•^OH is affected in a similar way, thus [^•^OH] is almost constant at pH 7–8 and decreases at pH > 8 (Figure 2a).

CO_3_^2−^ + ^3^CDOM* → CO_3_^•−^ + CDOM^•−^ (8)

The implications of the previously mentioned changes in [CO_3_^•−^] and [^•^OH] were investigated in the case of APAP photodegradation. As shown in Figure 1a, APAP is mainly degraded by CO_3_^•−^, while ^•^OH plays minor role. Furthermore, ^3^CDOM* and the direct photolysis of APAP are not much affected by pH [21].

As a consequence, the pH trend of *k*_APAP_ (Figure 2b) mirrors that of [CO_3_^•−^] (Figure 2a), so that *k*_APAP_ increases by ~5 times as pH increases from 6.5 to 9 at constant *C*_tot_ (note that photodegradation of DIC or NAP would be poorly affected in the same conditions).

The steady-state [CO_3_^•−^] is also heavily influenced by the dissolved organic carbon (DOC), which measures the content of dissolved organic matter (DOM) in water. Actually, DOM is able both to scavenge CO_3_^•−^ directly and to consume ^•^OH, thereby additionally inhibiting the formation of CO_3_^•−^ by reactions 1 and 2. Therefore, [CO_3_^•−^] is considerably inhibited by a DOC increase [11]. As shown in Figure 2b, an increase in DOC from 1.0 to 2.7 mg_C_ L^−1^ would, for instance, offset a pH increase from 6.5 to 9, thereby negating the pH-induced enhancement of APAP photodegradation. At the same time, a DOC decrease from 1.0 to 0.35 mg_C_ L^−1^ would offset a pH decrease from 9 down to 6.5.

**Figure 3:**
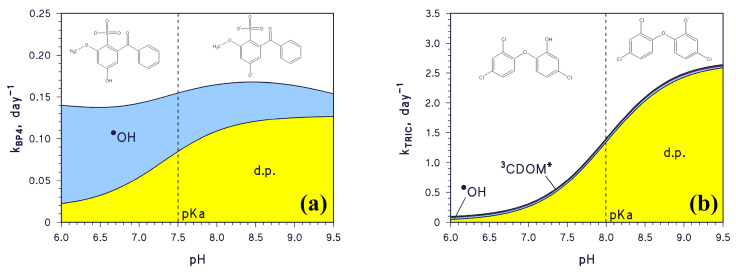
Trend with pH of the modelled first-order photodegradation rate constants of (**a**) BP4 (*k*_BP4_) and (**b**) TRIC (*k*_TRIC_). Water conditions: 3 m depth, 10^−4^ M NO_3_^−^, 10^−6^ M NO_2_^−^, *C*_tot_ = 10^−3^ M, DOC = 1 mg_C_ L^−1^, sunlight irradiance as per fair-weather spring equinox noon at mid latitude. The pKa values of BP4 and TRIC are highlighted on the plot (dashed vertical lines). Different transformation pathways are highlighted by different colours: ^•^OH, ^3^CDOM*, as well as d.p. = direct photolysis.

A change in pH would also affect the photodegradation of compounds undergoing acid⇆base equilibria. An example is the sunlight filter benzophenone-4 (2-hydroxy-4-methoxybenzophenone-5-sulphonic acid, hereinafter BP4), which has pKa~7.5 and mainly reacts with ^•^OH and by direct photolysis [21]. As shown in Figure 3a, the ^•^OH reaction would dominate the transformation of acidic BP4, while the same would occur with the direct photolysis for the basic form. Because the steady-state [^•^OH] decreases with increasing pH, as shown in Figure 2a, and because enhanced direct photolysis of basic BP4 largely offsets the [^•^OH] decrease, the pseudo-first-order rate constant *k*_BP4_ fluctuates around 0.15 day^−1^ in the pH interval 6.0–9.5 (Figure 3a; lifetime around 4.5 days). Another example is the antibacterial agent triclosan (5-chloro-2-(2,4-dichlorophenoxy)phenol, hereinafter TRIC), which has pKa~8 and reacts with ^•^OH, ^3^CDOM*, and by direct photolysis [21]. Figure 3b shows that TRIC photodegradation becomes faster as pH increases, because the basic TRIC form undergoes direct photolysis to a much higher extent than acidic TRIC.

CO_3_^•−^ is also affected by total inorganic carbon levels. An increase in the concentration of total inorganic carbon in surface waters can be the consequence of increasing levels of atmospheric CO_2_. This phenomenon enhances the weathering of rocks such as dolomite ((CaMg)(CO_3_)_2_), with dissolution of magnesium in the form of bicarbonate (reaction 9) [28]. CaCO_3_ is less likely to dissolve than the corresponding Mg salt, because it is less water-soluble and its solubility decreases with increasing water temperature, as a consequence of global warming [28]. It should also be observed that increasing temperature might induce the precipitation of CaCO_3_ from previously nearly saturated solutions, a phenomenon that occurs in many lakes during summer and actually decreases the total concentration of inorganic carbon [11]. However, the predicted average temperature increase over some decades (a few °C), while substantial [20], is much lower than the winter–summer temperature gradient (often >20 °C) [11,28]; for this reason, enhanced CaCO_3_ precipitation due to global warming would likely play a minor role and would be unable to offset enhanced weathering of carbonate rocks.

CaMg(CO_3_)_2 (s)_ + CO_2 (g)_ + H_2_O → CaCO_3 (s)_ + Mg^2+^ + 2 HCO_3_^−^ (9)

**Figure 4:**
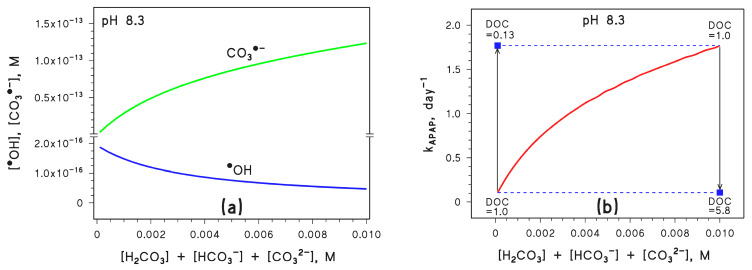
(**a**) Trend with *C*_tot_ = [H_2_CO_3_] + [HCO_3_^−^] + [CO_3_^2−^] of the steady-state [CO_3_^•−^] and [^•^OH]. (**b**) Trend with *C*_tot_ of the first-order photodegradation rate constant of APAP (*k*_APAP_); the DOC units are [mg_C_ L^−1^]. Conditions in both cases: 3 m depth, 10^−4^ M NO_3_^−^, 10^−6^ M NO_2_^−^, pH 8.3, DOC = 1 mg_C_ L^−1^ (unless otherwise stated), sunlight irradiance as per fair-weather spring equinox noon at mid latitude. Different curve colours are just for readability. The blue squares represent the impact of DOC variations on *k*_APAP_, the vertical arrows highlight the DOC changes, and the horizontal dashed lines are guides for the eye.

Reaction 9 accounts for the observed increasing levels of Mg^2+^, alkalinity, and inorganic carbon in river water [28]. If *C*_tot_ increases, [CO_3_^•−^] would increase because of enhanced formation of CO_3_^•−^ in reactions 1, 2, and 8. At the same time, [^•^OH] would decrease because of enhanced scavenging in reactions 1 and 2 (Figure 4a).

In a similar way, as in the case of pH, APAP photodegradation would follow the trend of [CO_3_^•−^] (Figure 4b) but DOC variations could offset changes in *C*_tot_ (increasing DOC would offset an increase in *C*_tot_ and vice versa).

Overall, Figure 2 and Figure 4 suggest that both [CO_3_^•−^] and *k*_APAP_ can be enhanced significantly by increasing pH and increasing *C*_tot_, provided that the DOC does not undergo a parallel increase that might be able to offset such variations. Actually, given the observed long-term changes of pH, inorganic carbon, and DOC in surface freshwaters [11,28], it can be inferred that a moderate DOC variation could more than compensate for quite large changes in pH and *C*_tot_. 

Differently, compounds affected by acid⇆base equilibria, such as BP4 and TRIC, could be more or less significantly impacted by pH changes, depending on their prevailing photodegradation pathways and on the relative photoreactivity of their acidic and basic forms toward each relevant pathway.

### 2.2. Photochemical Implications of Flow Changes in Rivers

Availability of water in rivers can fluctuate substantially as a consequence of flow variations, which can be induced, for instance, by a variable precipitation regime. In particular, climate change in many regions, such as the Mediterranean, may produce an alternation of extended drought periods and intense rain events [20], which would strongly affect river flow as a consequence [13].

This work has the goal of understanding the possible implications of these phenomena for photochemical reactions in river water. A decrease in the flow *Q* would likely cause river water to be shallower (lower depth *d*, which would enhance photochemical reaction kinetics), thinner (narrower river width, with no expected impact on photoreactions), and slower (lower velocity *U*) [11]. A decrease in *U* would increase the time that water takes to travel along the same river stretch, thereby providing more time for photochemical reactions to take place. Therefore, the combination of decreasing *d* and decreasing *U* is expected to enhance photochemical degradation reactions, because faster processes will also last for longer [29]. The opposite would understandably happen when *Q* increases.

**Figure 5:**
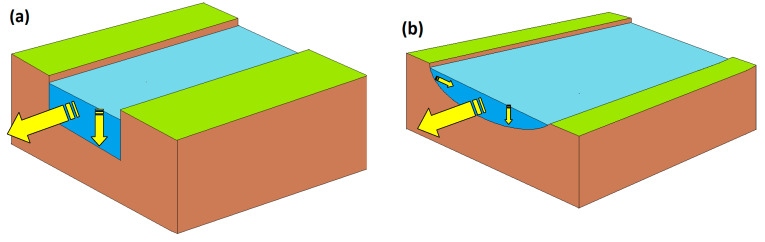
(**a**) Schematic of a channel with vertical slopes. In this case, a variation of the flow rate *Q* can only affect water velocity *U* and depth *d*, while width is geometrically bound to be constant. (**b**) In this watercourse, *U*, *d*, and width can all vary as *Q* is modified. Hereinafter, these two cases will be indicated as scenario (**a**) and scenario (**b**), respectively. Yellow arrows highlight water direction and changes in water level.

Quantitatively speaking, two possible scenarios can be hypothesised. The first (and the simplest) is a case resembling an artificial channel with vertical slopes (Figure 5a). Here, a variation in *Q* (m^3^ s^−1^ units) can be reasonably assumed to be evenly distributed between the only two dimensions/variables that can be modified, that is, depth *d* and velocity *U* (note that *Q* = *U* × *d* × *width*, but *width* is bound to be constant by the geometry of the system). In this case, given the initial values *Q*_o_, *U*_o_, and *d*_o_, it would be *d* = *d*_o_ (*Q*/*Q*_o_)^½^ and *U* = *U*_o_ (*Q*/*Q*_o_)^½^. In the second scenario (Figure 5b), a variation in *Q* would be distributed among all three dimensions (width, depth, and velocity). In a first-approximation hypothesis of an even distribution of such variation, it would be *d* = *d*_o_ (*Q*/*Q*_o_)^⅓^ and *U* = *U*_o_ (*Q*/*Q*_o_)^⅓^ (the analogous width variation would have practically no effect on photochemical reactions).

The impact of a variation in *d* on photoreaction kinetics can be captured by the APEX software [21], and it is reflected in modifications of the photodegradation rate constants (*k*) and half-life times (*t*_½_) of the contaminants. Briefly, photochemical reactions are faster in shallow waters that are better illuminated by sunlight compared to deep waters. The corresponding variations in *U* can be combined with those of *t*_½_ to define the length of the river stretch, which is required for the concentration of the contaminant to be halved by photochemical reactions: it is the half-life length *l*_½_ = *U t*_½_ [11]. On a relatively long river stretch, the values of *d* and *U* should be intended as averages because they both can vary (even at constant *Q*) if the watercourse becomes larger or narrower.

Operationally, we started by assuming initial conditions of *Q*_o_ = 100 m^3^ s^−1^, *U*_o_ = 1 m s^−1^ (reasonable for lowland rivers), and *d*_o_ = 5 m. In the case of a channel with vertical walls (scenario (a)), this result would be obtained with 20 m width. Assumed water-chemistry conditions were 10^−4^ M NO_3_^−^, 10^−6^ M NO_2_^−^, 10^−3^ M HCO_3_^−^, 10^−5^ M CO_3_^2−^, and 1 mg_C_ L^−1^ DOC. The APEX software was thus run with these chemistry and depth parameters, to obtain as output the *k* and *t*_½_ values for APAP, DIC, and NAP.

**Figure 6:**
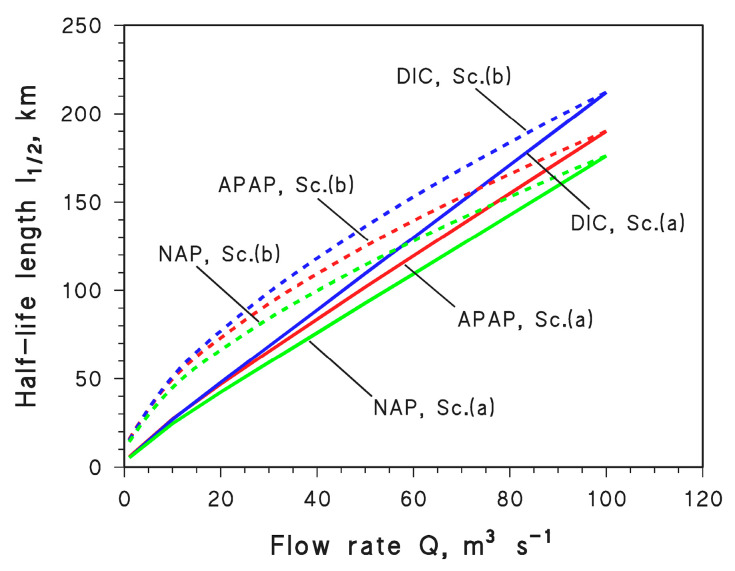
Trends of the half-life length *l*_½_ as a function of the flow rate *Q* for APAP, DIC, and NAP in scenario (a) (Sc. (a), solid curves) and scenario (b) (Sc. (b), dashed curves), respectively.

Afterwards, the value of *Q* was assumed to decrease from *Q*_o_ down to 10^−2^ *Q*_o_ at constant water chemistry, in the two scenarios where *d* = *d*_o_ (*Q*/*Q*_o_)^½^ (scenario (a)) and *d* = *d*_o_ (*Q*/*Q*_o_)^⅓^ (scenario (b)). In both cases, the *l*_½_ values of APAP, DIC, and NAP were determined from the values of *t*_½_ (calculated with APEX) that were multiplied by the associated values of *U* (*U* = *U*_o_ (*Q*/*Q*_o_)*^a^*, where *a* = ½ or ⅓). The results (*l*_½_ = *U t*_½_) are reported in Figure 6, and they show that *l*_½_ decreases as *Q* decreases. The *l*_½_ decrease is more marked for scenario (a), because modifications in *Q* affect two variables only (*U* and *d*), while in scenario (b) there is also an impact on water width that has no photochemistry implications. Therefore, *d* and *U* experience faster decrease with decreasing *Q* in scenario (a) than in scenario (b) and such decreases are transferred into *l*_½_.

Another interesting issue is that, although APAP, DIC, and NAP undergo photodegradation by different reaction pathways (Figure 1), *Q* has very similar effects on their photodegradation kinetics. Therefore, it can be assumed that changes in water flow affect to similar degrees the photoreactions of different CECs reacting via different pathways.

The half-life length *l*_½_, obtained as per the above procedure, can be used in a scenario that is based on the following, reasonable hypotheses: (i) The pollutant is emitted at one point along the river course, as in the case of a wastewater outlet, and undergoes quick mixing and dilution within the river-water flow *Q*. (ii) The wastewater flow is assumed to be constant while *Q* varies, which is reasonable because urban water consumption (and wastewater production as a consequence) has a daily trend but much smaller seasonal or long-term fluctuations compared to *Q* [29]. Therefore, the lower *Q* is, the lesser the dilution that the pollutant undergoes in river water becomes. (iii) The pollutant concentration decreases exponentially along the river course [29], and the extent of the decrease is measured by *l*_½_. It is thus possible to define ω = ln 2 (*l*_½_)^−1^ and, in analogy with an exponential decay over time, the concentration (*C*) trend of the pollutant would be the following:

*C* = *C*_o_ e^−ω *l*^ (10)

where *C*_o_ is the initial concentration of the pollutant soon after the point of emission (e.g., the wastewater outlet; note that quick—practically instantaneous—mixing is assumed here), and *l* is the distance travelled by water from the point of emission.

Note that, by so doing, ω is the equivalent of the rate constant *k* and *l* is the equivalent of time in the exponential decay.

**Figure 7:**
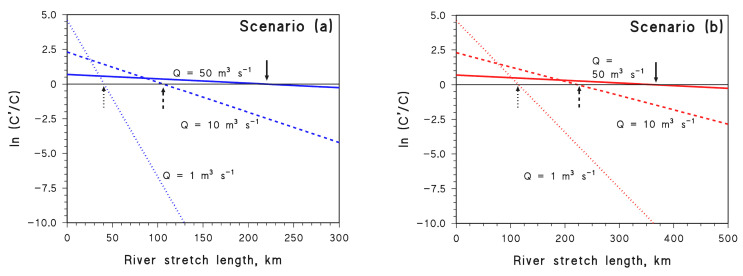
Trends of ln(*C*′/*C*) vs. *l* in the case of APAP, for scenarios (**a**) (*d* = *d*_o_ (*Q*/*Q*_o_)^½^, *U* = *U*_o_ (*Q*/*Q*_o_)^½^) and (**b**) (*d* = *d*_o_ (*Q*/*Q*_o_)^⅓^, *U* = *U*_o_ (*Q*/*Q*_o_)^⅓^). The arrows highlight the *l* values for which ln(*C*′/*C*) = 0 (i.e., *C*′ = *C*). Solid, dashed, and dotted lines are intended to highlight circumstances characterised by different values of *Q*.

Assume now that Equation (10) holds in the case of *Q*_o_. In the case of a different *Q* value, one has *C*′ = *C*′_o_ e^−ω′ *l*^. Combining the two equations and taking natural logarithms yields the following:

(11) $$\ln\frac{C^{\prime }}{C}=\ln\frac{{C^{\prime }}_{o}}{C_{o}}+(\omega -\omega^{\prime })\;l$$

where *C*′_o_/*C*_o_ = *Q*_o_/*Q* if the wastewater flow is much lower than the river flow. Moreover, if *Q* < *Q*_o_, the pollutant is initially more concentrated soon after the wastewater outlet, thus *C*′_o_ > *C*_o_. Plots of ln(*C*′/*C*) vs. *l* are shown in Figure 7 for different values of *Q*, in scenario (a) (Figure 7a) and scenario (b) (Figure 7b). The case of APAP is only shown here for simplicity but, because APAP has intermediate but similar behaviour compared to DIC and NAP (Figure 6), analogous trends would be obtained for the other two compounds. It can be seen that, if *Q* is lower, the pollutant is initially more concentrated, but it also undergoes faster degradation.

The values of *l* for which ln(*C*′/*C*) = 0 (i.e., *C*′ = *C*, meaning that faster photodegradation exactly offsets lower dilution) are highlighted by arrows on the plots. Interestingly, in both scenarios (a) and (b), such *l* values decrease as *Q* decreases from 50 to 1 m^3^ s^−1^. This means that, if *Q* is lower, it is *C*′ = *C* nearer to the wastewater outlet.

To make an instance in the case of scenario (a), with reference to *Q*_o_ = 100 m^3^ s^−1^, when *Q* = 50 m^3^ s^−1^ one has *C*′_o_ = 2 *C*_o_ because wastewater is diluted two times less. However, *C*′ decreases faster than *C* (ω’ = 5.5 × 10^−3^ km^−1^ vs. ω = 3.6 × 10^−3^ km^−1^) due to the combination of lower *d* and lower *U*. In this case, it takes around 220 km of river after the wastewater outlet to get *C*′ = *C* (Figure 7a). If *Q* = 1 m^3^ s^−1^, it is *C*′_o_ = 100 *C*_o_, but the decrease in *C*′ along the river is very fast (ω’ = 4.4 × 10^−2^ km^−1^), then *C*′ = *C* is reached after around 40 km. In the case of scenario (b), the acceleration of the reactions with decreasing *Q* is lower and a longer river stretch *l* is required to obtain *C*′ = *C* (Figure 7b).

The value of *l* for which *C*′ = *C* can be calculated from Equation (11) by assuming ln(*C*′/*C*) = 0, which yields the following:

(12) $$l=\frac{\ln(Q_{o}/Q)}{\omega^{\prime }-\omega }$$

**Figure 8:**
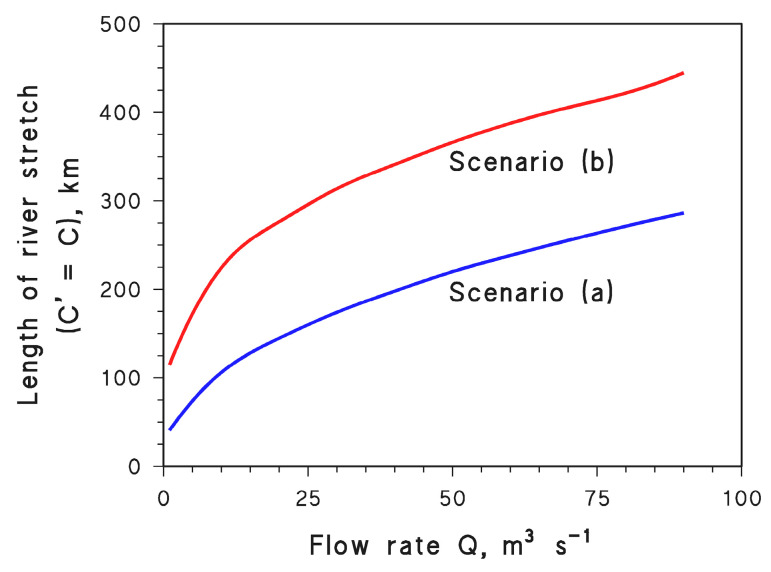
Values of *l* for which *C*′ = *C* (Equation (12)), as a function of the flow rate *Q* in the case of APAP in the two scenarios (scenario (a) and (b) as per Figure 5 and Figure 7).

The values of *l* vs. *Q* are reported in Figure 8 for both scenarios. On the one side, it appears that photochemical reactions have the potential to more than compensate for lower dilution of wastewater: the more severe the water scarcity, the shorter the value of *l* that gives *C*′ = *C*. 

However, it should also be considered that *C*′_o_/*C*_o_ = *Q*_o_/*Q*, which means that in case of severe water scarcity there is an initial stretch of river that is highly impacted by pollution, all the more so (higher *C*′_o_) as *Q* is lower.

Therefore, with *Q* = 1 m^3^ s^−1^ in scenario (a), on the one hand it is *C*′ = *C* after ~40 km and, for *l* > 40 km, the river would be even less polluted in the case of water scarcity than under normal flow conditions. On the other hand, however, one has *C*′_o_ = 100 *C*_o_ which means that, after the wastewater outlet, there is a relatively long river course that is highly impacted by pollution. This issue would be further exacerbated in scenario (b), where photochemical reactions are slower.

In summary, these results suggest that: (i) photochemical reactions are strongly enhanced during water scarcity, when they are key processes for the attenuation of many pollutants (and/or, if applicable, for the production of hazardous compounds [11]), but (ii) the same low-flow conditions that enhance photoreactions are also responsible for a considerable impact of pollutants on the water bodies, at least before photoinduced decontamination processes have had enough time to operate. Therefore, on the one hand, enhanced pollution impact due to lower dilution may potentially affect tens of kilometres of water downstream from a wastewater outlet. On the other hand, conditions would be much worse in the absence of photoreactions.

## 3. Methods

The APEX software (version 1.1) [21] was used for modelling the steady-state [^•^OH] and [CO_3_^•−^], as well as the photodegradation kinetics of APAP, DIC, NAP, BP4, and TRIC. As input data, APEX needs the spectral photon flux density of sunlight ([Ein cm^−2^ s^−1^ nm^−1^], a default one is available), water depth [m], the molar concentration values of NO_3_^−^, NO_2_^−^, HCO_3_^−^, and CO_3_^2−^, and the DOC [mg_C_ L^−1^] [30,31,32,33]. Furthermore, photodegradation kinetics parameters of the contaminant should be provided, including the direct photolysis quantum yield (Φ_dp_) plus the absorption spectrum, as well as the second-order reaction rate constants with ^•^OH, CO_3_^•−^, ^1^O_2_, and ^3^CDOM*. In the cases of APAP, DIC, NAP, BP4, and TRIC, the latter parameters are listed in Table 1.

For a given pollutant P, based on the above input data, APEX returns the steady-state concentrations [^•^OH], [CO_3_^•−^], [^1^O_2_], and [^3^CDOM*], as well as the pseudo-first-order photodegradation rate constant *k*_P_ ([day^−1^]; the software computes both the overall rate constant and those relevant to the separate photoreaction pathways). Such output data are here relevant to mid-latitude spring equinox conditions.

The pH value affects the concentrations [HCO_3_^−^] and [CO_3_^2−^], which can be obtained on the basis of pH and *C*_tot_ by means of Equations (6) and (7). Such equations thus played a key role in modelling the photochemical implications of pH and total carbonates. In the case of river flow changes, APEX can take into account the variations of *d*, i.e., the acceleration of photochemical reactions in shallower water columns. In contrast, calculations involving *U* and *l* had to be carried out as a posteriori on APEX output data.

## 4. Conclusions

Possible impacts of climate change on the chemistry and hydrology of surface waters in temperate environments have the potential to affect photodegradation processes and the photoinduced attenuation of contaminants. An increase either in pH (which might be caused by biological reduction of SO_4_^2−^, deriving from CaSO_4_ dissolution) or in the total carbonate concentration *C*_tot_ (weathering of dolomite by increased CO_2_) would enhance the steady-state [CO_3_^•−^] and decrease [^•^OH], thereby affecting the phototransformation of compounds that react with the two PPRIs. For instance, APAP photodegradation would be enhanced because this compound is mainly transformed upon reaction with CO_3_^•−^. However, for these effects to be highlighted, it is very important that changes in pH and *C*_tot_ take place at almost constant DOC, because relatively small DOC variations could easily offset/overcome important modifications of pH and *C*_tot_. Furthermore, compounds undergoing acid⇆base equilibria would be affected in different ways depending on the relative photoreactivity of their acidic and basic forms, as shown above for BP4 and TRIC.

The flow rate of rivers would be decreased by water scarcity phenomena, and lesser wastewater dilution would cause contaminants to reach higher concentration values downstream from wastewater treatment plants. At the same time, however, river water would be shallower and would flow more slowly, thereby enhancing the photochemical degradation of contaminants. In fact, photoprocesses are faster in shallower water that can be efficiently illuminated by sunlight, and they also have more time to take place as the water flows more slowly. Therefore, in the case of water scarcity, wastewater contaminants are initially more concentrated, but their concentration decrease is faster along the river stretch downstream from the wastewater outlet. Interestingly, the lesser the water flow, the shorter the river length needed for the low-flow contaminant concentration to reach the same value as the normal-flow concentration. After that point, the river would even be less contaminated in the low-flow scenario than in the normal-flow one but, if water flow is very low, there is a relatively long river stretch that is highly impacted by pollution before photodegradation can play significant role. Interestingly, photodegradation can be more efficient if water is not free to spread on the bed but, instead, the river is canalised or bound to pass between relatively narrow natural banks (scenario (a)).

A final issue is that lake and river waters might become increasingly oxygen-poor as a likely consequence of climate change [34,35]. The main implication of this phenomenon is an inhibition of processes triggered by ^1^O_2_ and a corresponding enhancement of reactions induced by ^3^CDOM*.

## Figures and Tables

**Table 1:** APEX input data concerning the photoreactivity of APAP, DIC, NAP, BP4 (both acidic and basic forms), and TRIC (same issue) [21]. “*Low*” means that the process has negligible importance.

	Φdp, mol Ein^−1^	kOH•, M^−1^ s^−1^	$k_{CO_{3}^{•-}}$, M^−1^ s^−1^	kCDOM∗3, M^−1^ s^−1^	kO21, M^−1^ s^−1^
APAP	4.6 × 10^−2^	1.9 × 10^9^	3.8 × 10^8^	1.6 × 10^9^	3.7 × 10^7^
DIC	9.4 × 10^−2^	9.3 × 10^9^	*Low*	6.4 × 10^8^	1.3 × 10^7^
NAP	1.0 × 10^−2^	8 × 10^9^	*Low*	7.5 × 10^8^	1.1 × 10^5^
BP4, acidic	3.2 × 10^−5^	1.9 × 10^10^	*Low*	*Low*	*Low*
BP4, basic	7.0 × 10^−5^	8.5 × 10^9^	*Low*	*Low*	*Low*
TRIC, acidic	0.3	5.4 × 10^9^	*Low*	3.1 × 10^9^	3 × 10^6^
TRIC, basic	0.3	1 × 10^10^	*Low*	4.3 × 10^9^	1.1 × 10^8^

## Data Availability

The data presented in this study are available on request from the corresponding author.
